# Lecture on Displacements of the Uterus

**Published:** 1839-06-15

**Authors:** W. Harris

**Affiliations:** At the Philadelphia Medical Institute


### CLINICAL LECTURE.

### LECTURE ON DISPLACEMENTS OF THE UTERUS, *delivered by* W. HARRIS, M. D., *at the Philadelphia Medical Institute.*

#### ANTEVERSION OF THE UTERUS.

ANTEVERSION is the opposite of retroversion. In this displacement the uterus lies horizontally in the antero-posterior direction of the pelvis, with the fundus towards the symphysis pubis, while the os tincæ is turned backwards and is high up in the hollow of the sacrum, pressing against the rectum.

As the direction of the uterus, in its natural situation, is parallel with the axis of the superior strait of the pelvis, which is an imaginary line extending from the umbilicus above, to the middle of the last bone of the sacrum below, it is evident that the natural inclination of the fundus uteri forwards, predisposes that organ to the displacement called anteversion, especially when the bladder is empty. And Madam Boivin says, in the third chapter of her work "On the Diseases of the Uterus," that anteversion is one of the most common displacements to which the uterus is subject, but that it is overlooked by physicians, "because it is most frequently complicated with some other affection of this organ, which exclusively occupies the attention."

Professor Moreau, on the contrary, observes that "anteversion takes place less frequently than retroversion, and that the unimpregnated uterus is more liable to this displacement than the gravid. In the latter state of the organ, its occurrence is excessively rare."

#### THE CAUSES OF ANTEVERSION.

The causes which produce this displacement are like those that produce retroversion, such as sudden and heavy falls, severe blows, violent vomiting, large pelves, injuries sustained in parturition, a morbid condition of the *utero-sacral* ligaments, travelling in carriages upon rough roads, long and fatiguing journeys, and, according to Levret, "a more than ordinary development and engorgement of the anterior parietes of the uterus itself." But this engorgement or hypertrophy of the anterior parietes, is, doubtless, entitled to be ranked among the effects rather than the causes of the mal-position, as the thickening is, according to the observations of Desormaux, "in proportion to the duration of the displacement." Moreover, in retroversion, when the fundus lies in the reverse direction, the posterior walls of the uterus have been found similarly hypertrophied.

Professor Moreau thinks that one of the most common predisposing causes of this mal-position, is an extraordinary degree of obliquity of the pelvis, in which the plane of the superior strait dips down anteriorly more than usual, and, consequently, there is a greater obliquity in the direction of the uterus, the axis of which corresponds with the axis of the abdominal strait. "The exciting causes," says he, "are nearly the same as those that produce retroversion; with this difference only, that in anteversion the displacing force must be exerted upon the posterior face of the uterus, in order to carry the fundus forwards and downwards, whilst in retroversion, the displacing impulse must be made in the opposite direction."

In the first case related by Levret, the displacement was produced suddenly by the patient falling upon her knees. In the two others which he relates, the displacement came on slowly, without any appreciable cause.

In Chopart's case, the displacement was occasioned in the second month of pregnancy, by vomiting. In another case, in a silk weaver, reported by Desgranges, it was the consequence of the repeated effort of pressing the foot upon the treadle of her loom. Madam Legrand's case of incurable anteversion, was the result of adhesions of the os uteri to the posterior parietes of the vagina. And Lehrbuch ascribed his case to an accumulation of fæces in the sigmoid flexure of the colon.

#### THE SYMPTOMS OF ANTEVERSION.

The patient afflicted with this complaint, experiences dull pains in the hypogastric region, and a sense of fulness or weight about the rectum. She has a frequent desire, moreover, to void her urine, which she always accomplishes with difficulty. The horizontal position affords her great comfort, and she is equally easy whether she lies upon her back or either side, if her limbs are flexed, so as to relax her abdominal muscles. But when she again resumes the erect position, she experiences the sensation of a body falling behind the pubes, which again occasions a retention of urine, and the same frequent desire to evacuate it. These symptoms have induced the attending physician to suspect the presence of stone in the bladder, and his suspicions have sometimes been strengthened by beholding the stream of urine suddenly arrested. And the urine itself, in old cases, becomes thick, reddish, sedimentous, and gravelly.

If a patient labouring under this displacement is unable to evacuate her urine out of bed, after she lies down upon her back, she will feel a body or weight retire from the bladder, when the emission of the urine will take place without much difficulty.

If a sound be introduced into the bladder, it meets a solid, resisting, but not a sonorous body, resembling the sensation afforded by an encysted stone or scirrhous tumour; but an examination, per vaginam, will dissipate all uncertainty. The fundus of the uterus will be discovered to have fallen forwards behind the symphysis pubis, while the os tincæ will be turned backwards towards the hollow of the sacrum near the sacro-vertebral angle. It is sometimes difficult to reach either the neck or mouth of the uterus through the vagina, but in such cases the diagnosis may be perfected through the rectum.

### THE TREATMENT.

The indications in the treatment are to restore the displaced organ, and, by appropriate means, to maintain it in its place.

The reduction is more easily accomplished than it is in retroversion.

The patient should be placed upon her back, with her breech projecting over the edge of the mattress, her thighs bent upon her body, and her legs upon her thighs, in order to relax the abdominal muscles. Her pelvis should be more elevated than her shoulders. The position is the same as that recommended in preternatural labour.

The operator should now introduce a large female sound into the bladder, and, afterwards, with the index finger of the right hand should reach the *os tincæ*, and, hooking it over the *anterior lip*, should draw it slowly from the hollow of the sacrum towards the symphysis pubis; at the same time, by means of the sound in the bladder, the *fundus uteri* should be pushed upwards and backwards to its natural position. An assistant may contribute to the restoration by making gentle pressure upon the hypogastrium, just above the symphysis pubis.

Having reduced the displaced organ, a pessary should be introduced, and the patient directed to lie upon her back for several days, after which she should, at first, walk about with care, lest any sudden jar or fall should occasion a return of the displacement.

The cup-in-ball (*bibloquet*) pessary, having a deep cavity to receive the cervix uteri, is the kind that is preferred in cases of anteversion. The common concave glass pessary will answer the purpose very well. This instrument should be applied, according to Levret, twelve or fifteen months, but Désormaux says it may be discontinued much sooner.

In every instance, the pessary produces more or less leucorrhœa, which diminishes, after it is worn for some months, and subsides, as soon as it is removed. If the patient should be much incommoded by fluor albus, and heat in the vagina, I am persuaded that the *perpetual douche*, invented by Professor Jackson, might be used with great advantage.

Whether the utero-abdominal supporter of Mrs. Betts, or Dr. Hull, would answer better in anteversion than the pessary, is yet to be proved.

A little reflection upon the subject must satisfy every unprejudiced mind, that the supporter meets exactly every indication in this displacement.

The celebrated Levret had the honour first to notice this displacement, nearly a century since, which he designated by the name of *renversement transversal.* The discovery was made by an *autopsy* of the body of a woman who died in consequence of an operation that had been performed upon her for a supposed encysted stone of the bladder.

Upon inquiry of her friends, after her death, Dr. Levret learned "that she was thirty years of age; that she had never menstruated; that about ten years before, she had a violent fall upon her knees, after which she was not able, without difficulty, to evacuate either her bladder or rectum, and that, although she never had had any children, she had often been thought to be pregnant, not only on account of the derangement of her catamenial function, but because of the pains which she experienced continually, more or less violent, in the lower part of her abdomen, especially when she was much in a standing position.

M. Levret was afterwards called, in consultation, by M. Lomain, to a patient whom he had previously sounded, and pronounced, as his judgment, that she had an encysted stone in her bladder. But when Levret heard the patient state, in answer to his enquiries, that "for twelve months immediately preceding, she had been much distressed with a dull pain in the hypogastrium, accompanied by an annoying sense of weight about the fundament and posterior parts of the pelvis, which were greatly exasperated when efforts were made use of to empty the contents of the bladder and rectum; and that when she assumed a standing position, she was apt to feel a heavy body, *as if falling down within her bladder*, which at the same time that it occasioned a desire to make water, deprived her of the power of obeying the call; but that when she lay down again, she felt the same body retire to its former situation, after which she could evacuate the contents of her bladder with less difficulty"—I repeat, that when Levret heard these facts stated, he drew the parallel between them and the history of the case of his former patient, and expressed his apprehension that this was another case of anteversion. M. Lomain now made a per vaginam examination, which he had not thought necessary to make before, when he found that Levret's apprehensions were well founded.

The organ was accordingly restored, the pessary introduced, and the patient eventually cured.

While wearing the pessary, this patient, we are told, "was much incommoded by a troublesome fluor albus," which, during the latter months of the treatment, diminished and subsided entirely after the instrument was withdrawn.

Levret attended a third case, in consultation with M. Couteveaux, in which the symptoms, treatment, and result, were nearly the same as in the second. In this case, however, the pessary was even more irritating, as "a troublesome leucorrhœal discharge, accompanied by a considerable sense of heat and smarting pains in the vaginal surfaces," followed its introduction.

Moreau relates a case to which he was called on the 4th of April, 1816, which, in its history, treatment, and result, resembles so nearly the second case of Levret, that a statement of it here would throw no new light upon our subject, neither would the several cases related by Madame Boivin and Madame Lachapelle.

To those who wish to investigate this subject more thoroughly, I would recommend for their perusal, the works of Davis, Moreau, and Levret, as well as those of Madame Boivin, and Madame Lachapelle.

